# Comparative Study of Time-Resolved Fluorescent Nanobeads, Quantum Dot Nanobeads and Quantum Dots as Labels in Fluorescence Immunochromatography for Detection of Aflatoxin B_1_ in Grains

**DOI:** 10.3390/biom10040575

**Published:** 2020-04-09

**Authors:** Xin Wang, Xuan Wu, Zhisong Lu, Xiaoqi Tao

**Affiliations:** 1 College of Food Science, Southwest University, Chongqing 400715, China; wx2018@email.swu.edu.cn; 2 Chongqing Animal Disease Prevention and Control Center, Chongqing 401120, China; lzpzena@swu.edu.cn; 3 Institute for Clean Energy & Advanced Materials, School of Materials & Energy, Southwest University, Chongqing 400715, China; zslu@swu.edu.cn

**Keywords:** time-resolved fluorescent nanobeads, quantum dot, quantum dot nanobeads, immunochromatography, aflatoxin B_1_

## Abstract

Label selection is an essential procedure for improving the sensitivity of fluorescence immunochromatography assays (FICAs). Under optimum conditions, time-resolved fluorescent nanobeads (TRFN), quantum dots nanobeads (QB) and quantum dots (QD)-based immunochromatography assays (TRFN-FICA, QB-FICA and QD-FICA) were systematically and comprehensively compared for the quantitative detection of aflatoxin B_1_ (AFB_1_) in six grains (corn, soybeans, sorghum, wheat, rice and oat). All three FICAs can be applied as rapid, cost-effective and convenient qualitative tools for onsite screening of AFB_1_; TRFN-FICA exhibits the best performance with the least immune reagent consumption, shortest immunoassay duration and lowest limit of detection (LOD). The LODs for TRFN-FICA, QB-FICA and QD-FICA are 0.04, 0.30 and 0.80 μg kg^−1^ in six grains, respectively. Recoveries range from 83.64% to 125.61% at fortified concentrations of LOD, 2LOD and 4LOD, with the coefficient of variation less than 10.0%. Analysis of 60 field grain samples by three FICAs is in accordance with that of LC-MS/MS, and TRFN-FICA obtained the best fit. In conclusion, TRFN-FICA is more suitable for quantitative detection of AFB_1_ in grains when the above factors are taken into consideration.

## 1. Introduction

Aflatoxin is a type of secondary metabolite produced mainly by microscopic fungal species *Aspergillus flavus* and *Aspergillus parasiticus* in the environment of high temperature and humidity (temperature 25–30 °C, moisture > 15%) [1]. According to the International Agency for Research on Cancer (IARC) [2], aflatoxins have been classified as a grade I carcinogenic substance. Among them, aflatoxin B_1_ (AFB_1_) is the most toxic, with strongest carcinogenicity; it contaminates more than 100 kinds of foods such as grain, oils, milk, condiments, nuts, tea and dairy products [3,4]. Since AFB_1_-caused food contamination comprises about 75% out of total mycotoxin contaminations [5], maximum residue limits (MRLs) for AFB_1_ in grains have been set (from 2 to 20 μg kg^−1^) in many countries, including the European Union (EU), the United States of America and China [6,7,8].

To better monitor the threat of AFB_1_ contamination, various methods have been developed in the past few decades [9,10,11,12]. Although the results are reliable and accurate, instrumental techniques [13] need expensive equipment and complicated sample pretreatment. Biosensors based on the antibody immunoprobes such as enzyme-linked immunosorbent assay (ELISA) [14] and fluorescence-linked immunosorbent assay (FLISA) [15,16] can achieve quantitative detection with good performance of specificity, sensitivity and simplicity, but the heterogeneous immunoassays require multiwashing procedures and long analysis times. To address the above issues, lateral flow immunochromatography assays have been considered as a promising method for onsite screening of mycotoxins [17,18,19]. Moreover, immunochromatography assays based on fluorescent markers (time-resolved fluorescent nanobeads (TRFN), quantum dot nanobeads (QB) and quantum dots (QD), etc.) have gradually become a popular research field in recent years for their advantages of sensitivity, accuracy, automated detection, shorter detection time, and so on [20,21,22]. Several fluorescence immunochromatography assays for highly sensitive detection of AFB_1_ have been reported [20,21,23,24,25].

**Figure 1:**
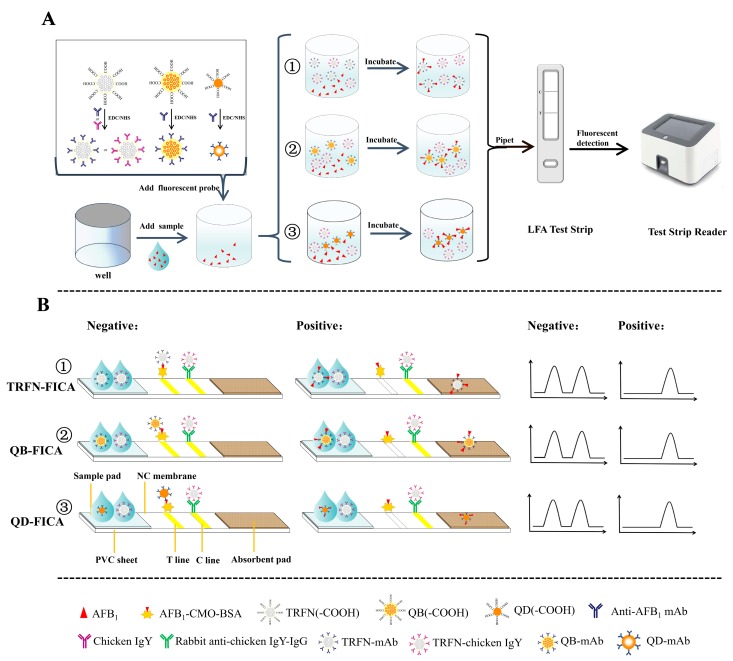
Schematic demonstration of (**A**) the procedures for aflatoxin B1 (AFB_1_) detection with fluorescence immunochromatography and (**B**) the principle of fluorescence immunochromatography assays for time-resolved fluorescent nanobeads (TRFN)-FICA, quantum dot nanobeads QB-(FICA) and quantum dots (QD)-FICA.

Although many methods based on immune interactions have been developed for the detection of toxic and harmful substances, it is impossible to compare the performance of those methods for identifying the most appropriate approach due to the utilization of distinct antibodies/antigens, markers and the detection conditions. In recent years, only a few reports have used comparative methods under the same conditions [26,27,28,29,30,31]. For instance, Xie et al. [27] established flow immunochromatography to detect *Escherichia coli O157:H7* in milk, in which fluorescent microspheres and colloidal gold were compared in terms of antibody labeling efficiency, sensitivity, antibody consumption and coefficient of variation. Wu et al. [28] systematically and comprehensively compared the performance of fluorescent microsphere and quantum dot immunochromatographic strips for quantitative detection of aflatoxin M_1_ (AFM_1_) in milk. However, to the best of our knowledge, among the widely used fluorescent labeling materials of TRFN, QB and QD, there are no clear statements on which labeling material is better for AFB_1_ detection in foods by immunochromatography. In this paper, in order to find a more suitable fluorescent detection method for quantitative detection of AFB_1_ in grains, TRFN, QB and QD were used as labels to establish fluorescent immunochromatography (TRFN-FICA, QB-FICA and QD-FICA) for the first time by comparing antibody labeling efficiency, detection sensitivity, antibody and antigen consumption, and accuracy under the same conditions (Figure 1).

## 2. Materials and Methods

### 2.1. Materials and Apparatus

#### 2.1.1. Materials

Time-resolved fluorescent nanobeads (TRFN, 1%, solid content, *w*/*v*; carboxylate-modified Eu (III)-chelate-doped polystyrene nanobeads; excitation = 365 nm, emission = 610 nm) were purchased from Bangs Laboratories, Inc. (Fishers, Hamilton, IN, USA). Carboxylated quantum dot nanobeads (QB, 1 uM, *w*/*v*, excitation = 365 nm, emission = 610 nm) and quantum dots (QD, 1.0 mg/mL, *w*/*v*; carboxylate-modified CdSe/ZnS core/shell nanocrystals with amphiphilic polymer coating; excitation = 365 nm, emission = 610 nm) were purchased from NanoGen (Beijing, China). Anti-AFB_1_ monoclonal antibody (mAb) and coating antigen (AFB_1_-CMO-BSA) were donated by Beijing WDWK Biotech Co., Ltd., (Beijing, China). N-hydroxysuccinimide (NHS) and 1-ethyl-3-[3-(dimethylamino) propyl] carbodiimide (EDC) were obtained from Aladdin (Shanghai, China). AFB_1_, aflatoxin B_2_ (AFB_2_), AFM_1_, aflatoxin M_2_ (AFM_2_), aflatoxin G_1_ (AFG_1_), aflatoxin G_2_ (AFG_2_), zearalenone (ZEN), ochratoxin A (OTA), deoxynivalenol (DON) and bovine serum albumin (BSA) were purchased from Sigma (St. Louis, MO, USA). Chicken IgY and rabbit antichicken IgY-IgG were obtained from Biodragon Immunotechnologies Co., Ltd. (Beijing, China). Other chemical substances were purchased from Beijing Chemical Reagent Company (Beijing, China). All solvents and other chemicals were of analytical reagent grade and did not require further purification. A working standard of AFB_1_ was prepared from the 2 mg mL^−1^ stock solution by serial dilution with a sample buffer solution (0.3 M Tris-HCl containing 0.5% polyvinyl pyrrolidone and 0.4% Tetronic 1307, pH 8.0). 

The nitrocellulose (NC) membrane (Unistart CN95) was acquired from Sartorius Stedim Biotech GmbH (Goettingen, Germany). The sample pad (glass fiber) and the absorbent pad were supplied by Shanghai Liangxin Co., Ltd. (Shanghai, China). The microtiter plates were supplied by Guangzhou JET BIOFIL Co., Ltd. (Guangzhou, China).

#### 2.1.2. Apparatus

An XYZ3060 dispensing platform was purchased from Bio Dot Inc. (Irvine, CA, USA). The CM4000 guillotine-cutting module was purchased from Kinbio Tech Co., Ltd. (Shanghai, China). The fluorescence immunochromatography quantitative analyzer was purchased from WDWK Bio Co., Ltd. (Beijing, China). Ultrapure water was purified with the Milli-Q system from Millipore Corp. (Bedford, MA, USA). The size distributions and surface morphologies of the three labels were determined by transmission electron microscope (JEM 1200EX, Tokyo, Japan). The mAb labels were characterized with a particle size analyzer (Malvern Instruments Ltd., Worcestershire, UK).

### 2.2. Preparation of Three Labeled Antibody Probes

The TRFN-mAb was prepared based on the procedures described in the previous literature with slight modification [26,30]. Briefly, 5 μL of TRFN was dissolved in 45 μL of activation buffer (50 mM MES (2-Morpholinoethanesulfonic Acid), pH 6.0) and then centrifuged at 20,000× *g* for 15 min at 4 °C. Subsequently, 40 μL of activation buffer, 5 μL of NHS solution (1 mM) and 5 μL of EDC solution (1 mM) were added to the tube and stirred for 15 min; the solution was centrifuged at 20,000× *g* for 15 min and the precipitate was resuspended in 25 μL boric acid buffer (40 mM, pH 8.0). Next, 25 μL of anti-AFB_1_-mAb was added to the suspension and incubated at room temperature for 2 h, then centrifuged, and the precipitate was dissolved in 100 μL of blocking buffer (20 mM PBS, 50 mM ethanolamine, 4% BSA, pH 7.4) for 2 h. After the end of the blocking, the mixture was centrifuged at 20,000× *g* for 15 min at 4 °C, and the precipitate was resuspended in 50 μL of complex solution (10 mM Tris, 1% BSA, 2% sucrose, 2% trehalose, pH 8.5) at 4 °C until use. Ultrasonic dispersion was required for 3 min after each resuspension by centrifugation.

The preparation of TRFN-IgY, QB-mAb and QD-mAb probes was identical to the preparation of TRFN-mAb, and the only differences were that chicken IgY was used instead of anti-AFB_1_-mAb, and QB and QD were used instead of TRFN, respectively. All the labeled antibody probes were stored at 4 °C until use.

### 2.3. Preparation of the Fluorescence Immunochromatography Assay Strips

The fluorescence quantitative immunochromatographic strips consisted of four parts: absorbent pad, NC membrane, sample pad and adhesive plastic-backing sheet (Figure 1B). The procedures for making test strips were the same as our previously reported work with some modifications [32,33]. Briefly, a proper amount of AFB_1_-CMO-BSA and rabbit antichicken IgY-IgG were separately sprayed onto the NC membrane as capture reagents to form *T* line and *C* line. The distance between *T* and *C* line was 1.2 cm and the dispense rate was 0.7 μL cm^−1^. Afterward, the dried NC membrane, sample pad and absorbent pad were laminated and cut into 4.7 mm wide test strips. Finally, the PVC sheet and strip were installed onto a plastic plate and stored in dry conditions at 4 °C until use.

### 2.4. Sample Preparation and Detection

The sample preparation procedure was applied for corn, soybeans, sorghum, wheat, rice and oats. First, all samples were ground into powder and sieved through 20 mesh; then 1.00 ± 0.05 g of the pulverized samples were extracted with 4 mL of methanol/water solution (70/30, *v*/*v*); the mixture was vortexed for 5 min and centrifuged at 4000× *g* for 5 min at room temperature. Afterwards, 1 mL of the supernatant was diluted with 9 mL of sample buffer solution (0.3 M Tris-HCl containing 0.5% polyvinyl pyrrolidone and 0.4% Tetronic 1307, pH 8.0) to obtain a sample treatment solution. 

Finally, an appropriate amount of fluorescent probes was added and incubated with 120 μL of sample treatment solution for 5 min at room temperature (25 °C) in the microwell; 85 μL of incubated working solution was added into the test area. The fluorescence intensity ratio of *T* line and *C* line were defined as *F_T_* and *F_C_*. The fluorescence values of *F_T_*, *F_C_* and *F_T_*/*F_C_* were collected for quantification.

### 2.5. Establishment of Quantitative Calibration Curves

The quantitative calibration curves were established by plotting *B/B_0_* (the concentration of the analyte was 0 μg L^−1^, the value of *F_T_*/*F_C_* was marked as *B*_0_; while the concentration of the analyte was at other concentrations, the value of *F_T_*/*F_C_* was marked as *B*) against the logarithm of AFB_1_ concentration. Different concentrations of AFB_1_ (0, 5 × 10^−4^, 1 × 10^−3^, 5 × 10^−3^, 0.01, 0.05, 0.1, 0.5 and 1 μg L^−1^) were prepared by diluting in sample buffer solution; each piece of data was repeated for 6 times and fit to a four-parameter logistic equation using Origin (version 8.5, OriginLab, USA) software packages,

$$y=\left(A-D\right)/\left[1+{\left(x/C\right)}^{B}\right]+D$$

where A is the response value at high asymptote, B is the slope at the inflection point, C is the x value at the inflection point (corresponding to concentration resulting in 50% inhibition), D is the response value at low asymptote.

### 2.6. Validation of FICAs

For validation of TRFN-FICA, QB-FICA and QD-FICA, 60 different field grain samples (10 samples for each of corn, soybeans, sorghum, wheat, rice and oats) were analyzed by the three FICAs and liquid chromatography–tandem mass spectrometry (LC-MS/MS); the LC-MS/MS procedures were performed according to the standard method of “GB5009.22-2016” [34]. The detection performances of the three FICAs were compared to that of the LC-MS/MS to assess reliability. The LOD was calculated as the mean value of 20 blank samples plus three times the standard deviation (mean + 3SD). The accuracy of the method was investigated by spiking blank samples with single or multiple analytes at three concentrations (LOD, 2LOD, 4LOD). The recovery was calculated by the following equation: Recovery (%) = (measured concentration / fortified concentration) × 100%. The intra-assay and interassay precisions were represented by the coefficient of variation (CV); each sample was tested 6 times in duplicate and on three consecutive days.

## 3. Results and Discussion 

### 3.1. Principle of Three Fluorescence Labels for Detection of Aflatoxin B_1_

Three fluorescence labels were selected for the determination of AFB_1_ by direct competition reaction in general (Figure 1). Specifically, the rabbit antichicken IgY-IgG was immobilized on *C* line, and exhibited a constant *C* line fluorescence signal since the independent TRFN-chicken IgY was specially prepared for it. AFB_1_-CMO-BSA (coating antigen) was immobilized on *T* line, and when the fluorescence probes (QD-mAb, QB-mAb and TRFN-mAb) were not bound to free AFB_1_ molecules, they could be specifically captured by coating antigen as a reference signal in FICAs; otherwise, it would flow past both *T* and *C* lines with no signal. According to this principle, the adopted dual system (independent *T* and *C* lines) can maintain a comparatively stable *C* line fluorescence intensity with no interference; the fluorescence intensity of *T* line decreased with increased concentration of AFB_1_. Compared with previous studies of coating secondary antibodies to form *T* line [35,36], this dual system achieved better performance and could be applied in later reported immunochromatographic assays [37]. Overall, quantitative relationships can be established between the concentrations of AFB_1_ and *F_T_/F_C_* ratios, and can be further quantitatively calculated by the portable reader.

### 3.2. Characterization of Fluorescence Labels

**Figure 2:**
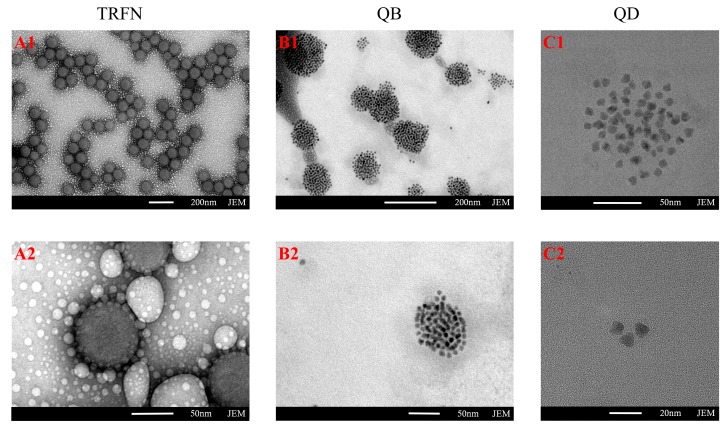
Size characterization of three labels: (**A1**,**A2**) TEM images of TRFN at 200 and 50 nm magnifications; (**B1**,**B2**) TEM images of QB at 200 and 50 nm magnifications; (**C1**,**C2)** TEM images of QD at 50 and 20 nm magnifications.

The surface morphology and size of the three labels (TRFN, QB, QD) were characterized by transmission electron microscope (TEM), showing that TRFN, QB, QD had relatively uniform size distribution (Figure 2). TRFN are composed of rare earth lanthanide chelates (such as Eu(III), Tb(III) and Dy(III)) and exhibit longer (microsecond) lifetimes, allowing fluorescence decay to be monitored over time. This technique provides a means to separate the “true” fluorescence signal from short-lived background fluorescence, and an opportunity to improve assay sensitivity [38]. QD are new fluorescent labels with great prospects, and have been widely used to improve the detection sensitivity of FICA because of their narrow emission spectra, broad excitation range and highly fluorescent quantum yields [20]. Furthermore, QB are tens of thousands of quantum dots wrapped in inorganic materials such as silicon dioxide by self-assembly, which is easy to mass produce; they have stronger fluorescence stability and intensity than the corresponding single QD [28]. These labels were distributed uniformly in the low magnification image and scattered well in the magnified view; the magnified TEM image in Figure 2(B2) revealed that the single quantum dots were embedded uniformly when compared to Figure 2(C2). After chemically binding to the surfaces of the antibody, these fluorescence labels provided a high degree of long-term stability in sample detection [28], and the particle size analyzer indicated that the average hydrodynamic diameters of TRFN-mAb, QB-mAb and QD-mAb were significantly increased from 90 (TRFN) to 113 nm, 110 (QB) to 136 nm, and 15 (QD) to 42 nm (Figure S1), respectively. This proved that the three fluorescent probes were successfully synthesized, and all the probes were used for fluorescence immunochromatography detection.

### 3.3. Optimization and Establishment of Standard Calibration Curve

To achieve the best performance of FICAs, parameters such as coupling pH, lateral flow immune response time, working concentration of labeled mAb immunoprobes (anti-AFB_1_-mAb) and working concentration of coating antigens (AFB_1_-CMO-BSA) were taken into consideration as important factors that affected the sensitivity of the FICAs. Therefore, all the FICAs needed to be introduced at optimum parameters. In this assay, the fluorescence intensity of *C* line was almost constant under the same reaction conditions (1.6 μg mL^−1^ of rabbit anti-chicken IgY-IgG as coating antigen and 3.4 μg mL^−1^ of TRFN-IgY as immunoprobe). The competitive inhibition ratio was observed by investigating appropriate fluorescence intensity of *T* line and *C* line, which was chosen as a factor to reflect the sensitivity of FICAs. As seen in Figure S2A, the fluorescence intensity of TRFN-FICA was enhanced with an increase of pH, and the highest competitive inhibition ratio was observed at pH 7.0; therefore, pH 7.0 was regarded as the optimal pH for coupling with TRFN. Using the same reasoning, pH 6.0 and 7.0 were the optimal labeling pH for QB-mAb and QD-mAb, respectively (Figure S2). In this study, the concentration parameters of labeled mAb immunoprobes (anti-AFB_1_-mAb) were 3.0, 4.5 and 4.5 μg mL^−1^ for TRFN-FICA, QB-FICA and QD-FICA, respectively (Figure S3). Coating antigens (AFB_1_-CMO-BSA) were 0.3, 0.65 and 0.65 μg mL^−1^ for TRFN-FICA, QB-FICA and QD-FICA, respectively (Figure S4). The optimum immunochromatography durations were 25, 30 and 35 min for TRFN-FICA, QD-FICA and QB-FICA, respectively (Figure S5).

### 3.4. Validation of FICAs

#### 3.4.1. Sensitivity

**Figure 3:**
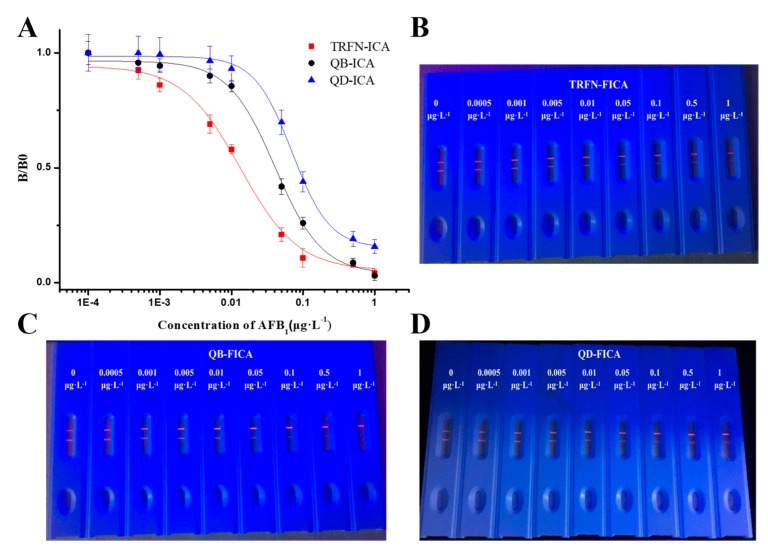
(**A**) Standard curves of TRFN-FICA, QB-FICA and QD-FICA for AFB_1_ and (**B**–**D**) corresponding immunochromatographic strips.

Under optimum conditions, with the increasing concentration of AFB_1_ diluted in sample buffer solution, the fluorescence intensity of the corresponding test line gradually decreased. The calibration curves of three fluorescent label-based FICAs were constructed by plotting *B*/*B*_0_ against the logarithm of AFB_1_ concentrations (Figure 3); we then fit the data using linear equations. The sensitivity of TRFN-FICA, QB-FICA and QD-FICA were evaluated using the values of IC_50_ obtained from the calibration curves, which were 0.0133, 0.0442 and 0.0848 μg L^−1^, respectively. The dynamic linear ranges, determined as the concentrations causing 20%–80% inhibition of *B*/*B*_0_, were 0.00368–0.04804, 0.01621–0.09775 and 0.03756–0.16776 μg L^−1^, respectively.

#### 3.4.2. Specificity 

To examine the specificity of proposed FICAs, three structurally-related mycotoxins, such as AFB_2_, AFM_1_ and AFG_1_, and non-structurally-related mycotoxins, including DON, OTA and ZEN, were tested individually by the FICAs to evaluate specificity (Table S1). Data were obtained from six replicates. All results clearly demonstrated that the three FICAs (TRFN-FICA, QB-FICA and QD-FICA) have negligible cross reactivity (CR < 20%) with the other mycotoxins, and the proposed three FICAs can be applied to detect AFB_1_ with high specificity.

### 3.5. Application to Grain Samples

Detection performance of the three FICAs was investigated in real samples. The LOD was calculated as the mean value of 20 blank grain samples plus three times the standard deviation (mean + 3SD). Each of the 20 blank grain samples (corn, soybean, sorghum, wheat, rice and oats) were extracted and analyzed according to the sample preparation and detection procedure. The LODs for TRFN-FICA, QB-FICA and QD-FICA were 0.04, 0.30 and 0.80 μg kg^−1^, respectively. TRFN-FICA possessed the advantages of sensitivity, rapidity, antibody and antigen consumption, and accuracy when compared with QB-FICA and QD-FICA (Table 1).

Moreover, in comparison with most available immunoassay methods for comprehensive performance (Table 2), the detected performances of QD-FICA and QB-FICA were in accordance with the reported fluorescence immunochromatography in real samples or buffer solution [23,39]; TRFN-FICA had the best LOD and reached 125%–150% better sensitivity than the reported multiple time-resolved fluorescence immunochromatography assay [21,24]. Therefore, fluorescence immunochromatography assay, especially TRFN-FICA, possessed the obvious advantages of sensitivity, rapidity and cost-effectiveness for onsite screening of AFB_1_ in grains [36,40,41,42].

**Figure 4:**
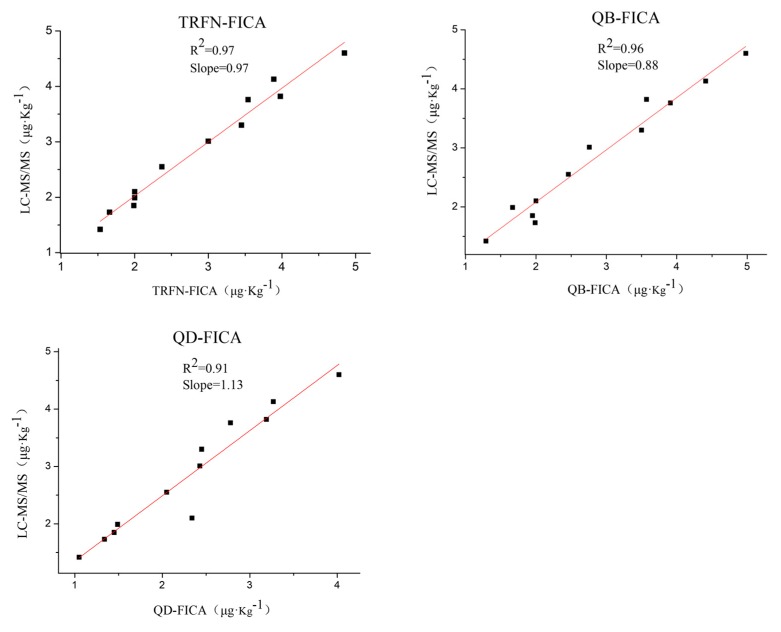
Consistent results between LC-MS/MS and the three FICAs (TRFN-FICA, QB-FICA and QD-FICA) in positive grain samples.

Furthermore, in order to verify and compare the reliability of FICAs, 60 grain samples were analyzed by liquid chromatography–tandem mass spectrometry (LC-MS/MS) [34], TRFN-FICA, QB-FICA and QD-FICA. A total of 12 samples were confirmed as positive samples, while others (48 samples) were negative by LC-MS/MS and three FICAs, and the representative mass chromatograms (highest and lowest concentrations for positive samples) are listed in Figure S6. There were no false negative or false positive results reported by the three FICAs, and analysis of field grain samples by FICAs were in accordance with that of LC-MS/MS (Figure 4). These results indicate that all three FICAs are reliable methods for the determination of AFB_1_ residues in grains, and that TRFN-FICA obtained the best fit.

### 3.6. Accuracy and Precision of Three Label-Based FICAs

**Figure 5:**
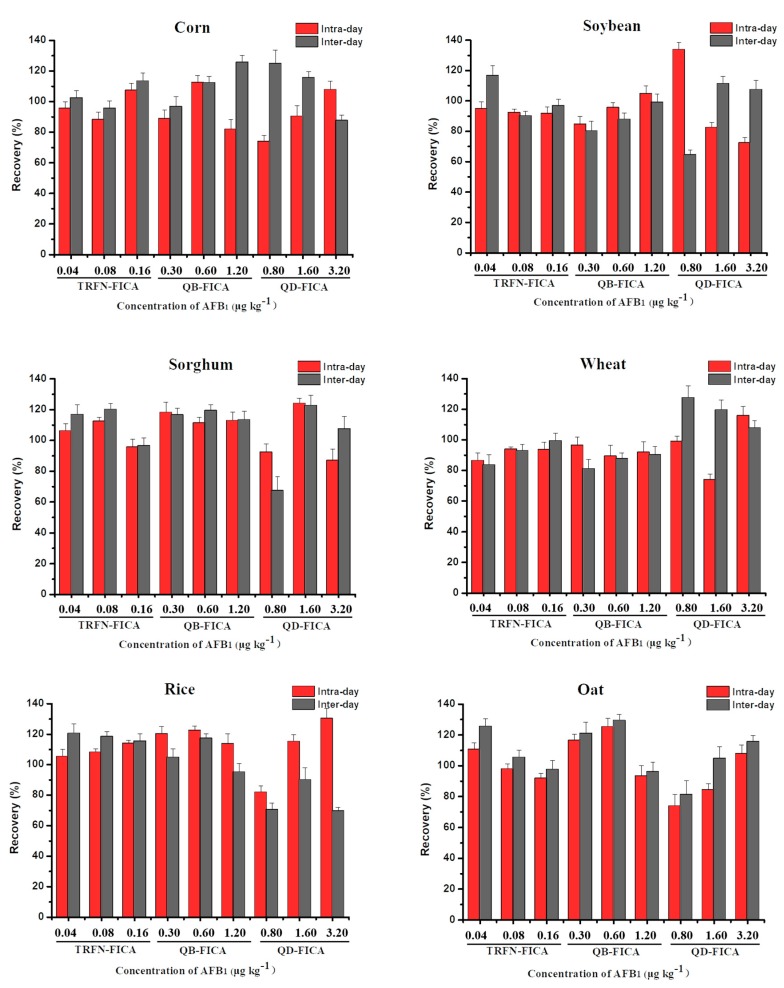
The accuracy and precision of TRFN-FICA, QB-FICA and QD-FICA in AFB_1_ in spiked samples.

We performed recovery experiments to assess the accuracy and precision of the three FICAs using six kinds of blank grain samples (corn, soybeans, sorghum, wheat, rice and oat) with a series of known concentrations of AFB_1_. The choices of low, medium and high concentrations with AFB_1_ were the same as in the previous reported literature [32,33], which were represented by LOD, 2LOD and 4LOD, respectively. Data were obtained from six replicates and on three consecutive days. The intraday and interday recovery of TRFN-FICA ranged from 86.48% to 114.10% and 83.64% to 125.61%, respectively; the coefficient of variation were all less than 10%. Meanwhile, TRFN-FICA had better recovery than QB-FICA and QD-FICA (Figure 5), confirming that the accuracy and precision of TRFN-FICA were better than QB-FICA and QD-FICA.

## 4. Conclusions 

In this study, three FICAs (TRFN-FICA, QB-FICA and QD-FICA) were systematically compared for the quantitative detection of AFB_1_ in grains successfully. Under optimum conditions, six types of grain samples were analyzed, showing that TRFN-FICA was the most consistent with LC-MS/MS. Moreover, TRFN-FICA had the lowest LOD, shortest immune duration (25 min), and less coating antigen consumption (0.30 μg) and antibody consumption (0.015 μg). Overall, compared with QB-FICA and QD-FICA, TRFN-FICA had a unique advantage in quantitative detection of AFB_1_ in grain, providing a reference for the selection of markers in detection methods.

## Figures and Tables

**Table 1:** Performance of TRFN-FICA, QB-FICA and QD-FICA in 6 grains.

Parameter	TRFN-FICA	QB-FICA	QD-FICA
LOD (μg kg^−1^)	0.04	0.30	0.80
Antibody usage per test card (μg)	0.015	0.09	0.03
The best coating for AFB_1_-CMO-BSA (μg)	0.30	0.65	0.65
Immunoassay duration (min)	25	30	35
Recovery (%)	83.64%–125.61%	80.29%–129.45%	64.53%–133.86%
Coefficient of variation (%)	3.10%–6.75%	2.88%–7.16%	2.34%–8.96%

**Table 2:** Comparison of immunoassays for determination of AFB_1_.

Detection Method	Marker	Target Substance	Sample	Detection Limit of AFB_1_ (μg kg^−1^)
Immunoadsorption [15]	Enzyme	AFB_1_	Feed samples	2.0
Multiplex immunochromatography [36]	Colloidal gold	AFB_1_, ZEN, OTA	Corn	0.10
			Rice	0.12
Fluorescent immunochromatography [24]	TRFN	AFB_1_	Corn	0.06
Fluorescent immunochromatography [20]	QB	AFB_1_	Buffer solution	0.005 (When the inhibition is 10)
Multiplex fluorescent immunochromatography [23]	QB	AFB_1_, ZEN	Buffer solution	0.00165 (When the inhibition is 10%)
Multiplex fluorescent immunochromatography [21]	TRFN	AFB_1_, ZEN	Buffer solution	0.05
Fluorescence resonance energy transfer [39]	QD	AFB_1_	Rice	0.04
Fluorescent immunochromatography (this study)	TRFN	AFB_1_	Corn, soybean, sorghum, wheat, rice and oats	0.04
	QB			0.30
	QD			0.80

## Supplementary Materials

The following are available online at https://www.mdpi.com/2218-273X/10/4/575/s1. Figure S1: Particle size of three FICA labels. Figure S2: Optimization of coupling pH in preparing labeled mAb probes: (A) TRFN-mAb; (B) QB-mAb; (C) QD-mAb. Figure S3: Optimization of the concentration of anti-AFB_1_-mAb in preparing labeled mAb probes: (A) TRFN-mAb; (B) QB-mAb; (C) QD-mAb. Figure S4: Optimization of the concentration of AFB_1_-CMO-BSA for fluorescent immunochromatography: (A) TRFN-FICA; (B) QB-FICA; (C) QD-FICA. Figure S5: Immunoreaction dynamics of the three FICAs: (A) TRFN-FICA; (B) QB-FICA; (C) QD-FICA. Table S1: Cross reactivity (CR) of analytes with antibody detected by FICAs.

## References

[1] Sun L.L., Zhao Q. (2018). Competitive horseradish peroxidase-linked aptamer assay for sensitive detection of Aflatoxin B1. Talanta.

[2] IARC Working Group (1993). IARC Monographs on the Evaluation of Carcinogenic Risks to Humans. v. 56: Some Naturally Occuring Substances: Food Items and Constituents, Heterocyclic Aromatic Amines and Mycotoxins.

[3] Jia Y.M., Zhou G.H., Liu P.L., Li Z.G., Yu B. (2019). Recent Development of Aptamer Sensors for the Quantification of Aflatoxin B1. Appl. Sci..

[4] Neagu D., Capodilupo A., Vilkanauskyte A., Micheli L., Palleschi G., Moscone D. (2009). AFB1-AP Conjugate for Enzyme Immunoassay of Aflatoxin B1 in Corn Samples. Anal. Lett..

[5] Danesh N.M., Bostan H.B., Abnous K., Ramezani M., Youssefi K., Taghdisi S.M., Karimi G. (2018). Ultrasensitive detection of aflatoxin B1 and its major metabolite aflatoxin M1 using aptasensors: A review. TrAC Trends Anal. Chem..

[6] Wei T., Ren P., Huang L., Ouyang Z., Wang Z., Kong X., Li T., Yin Y., Wu Y., He Q. (2019). Simultaneous detection of aflatoxin B1, ochratoxin A, zearalenone and deoxynivalenol in corn and wheat using surface plasmon resonance. Food Chem..

[7] (2017). GB 2671-2017. National Food Safety Standard for Mycotoxins Limits in Food.

[8] (2004). Compliance Policy Guides.

[9] Edupuganti S.R., Edupuganti O.P., Hearty S., O’Kennedy R. (2013). A highly stable, sensitive, regenerable and rapid immunoassay for detecting aflatoxin B1 in corn incorporating covalent AFB1 immobilization and a recombinant Fab antibody. Talanta.

[10] Ben Abdallah Z., Grauby-Heywang C., Beven L., Cassagnere S., Moroté F., Maillard E., Sghaier H., Cohen Bouhacina T. (2019). Development of an ultrasensitive label-free immunosensor for fungal aflatoxin B1 detection. Biochem. Eng. J..

[11] Eom T., Cho H.D., Kim J., Park M., An J., Kim M., Kim S.H., Han S.B. (2017). Multiclass mycotoxin analysis in edible oils using a simple solvent extraction method and liquid chromatography with tandem mass spectrometry. Food Addit. Contam. A.

[12] Xia X., Wang Y., Yang H., Dong Y., Zhang K., Lu Y., Deng R., He Q. (2019). Enzyme-free amplified and ultrafast detection of aflatoxin B1 using dual-terminal proximity aptamer probes. Food Chem..

[13] Huertas Perez J.F., Arroyo Manzanares N., Hitzler D., Castro Guerrero F.G., Gamiz Gracia L., Garcia Campana A.M. (2018). Simple determination of aflatoxins in rice by ultra-high performance liquid chromatography coupled to chemical post-column derivatization and fluorescence detection. Food Chem..

[14] Kolosova A.Y., Shim W.B., Yang Z.Y., Eremin S.A., Chung D.H. (2006). Direct competitive ELISA based on a monoclonal antibody for detection of aflatoxin B1 Stabilization of ELISA kit components and application to grain samples. Anal. Bioanal. Chem..

[15] Wei J.Y., Zhang D., Zhang L.X., Ouyang H., Fu Z.F. (2019). Alkaline Hydrolysis Behavior of Metal-Organic Frameworks NH2-MIL-53(Al) Employed for Sensitive Immunoassay via Releasing Fluorescent Molecules. ACS Appl. Mater. Interfaces.

[16] Beloglazova N.V., Speranskaya E.S., Wu A., Wang Z., Sanders M., Goftman V.V., Zhang D., Goryacheva I.Y., De Saeger S. (2014). Novel multiplex fluorescent immunoassays based on quantum dot nanolabels for mycotoxins determination. Biosens. Bioelectron..

[17] Posthuma-Trumpie G.A., Korf J., van Amerongen A. (2009). Lateral flow (immuno) assay: Its strengths, weaknesses, opportunities and threats. A literature survey. Anal. Bioanal. Chem..

[18] Dzantiev B.B., Byzova N.A., Urusov A.E., Zherdev A.V. (2014). Immunochromatographic methods in food analysis. TrAC Trends Anal. Chem..

[19] Anfossi L., Baggiani C., Giovannoli C., D’Arco G., Giraudi G. (2013). Lateral-flow immunoassays for mycotoxins and phycotoxins: A review. Anal. Bioanal. Chem..

[20] Li J.Y., Mao M., Wu F., Li Q., Wei L.Y., Ma L. (2018). Amino-functionalized CdSe/ZnS quantum dot-based lateral flow immunoassay for sensitive detection of aflatoxin B1. Anal. Methods.

[21] Tang X.Q., Li P.W., Zhang Q., Zhang Z.W., Zhang W., Jiang J. (2017). Time-Resolved Fluorescence Immunochromatographic Assay Developed Using Two Idiotypic Nanobodies for Rapid, Quantitative, and Simultaneous Detection of Aflatoxin and Zearalenone in Maize and Its Products. Anal. Chem..

[22] Wang Y.K., Shi Y.B., Zou Q., Sun J.H., Chen Z.F., Wang H.A., Li S.Q., Yan Y.X. (2013). Development of a rapid and simultaneous immunochromatographic assay for the determination of zearalenone and fumonisin B1 in corn, wheat and feedstuff samples. Food Control.

[23] Shao Y.N., Duan H., Guo L., Leng Y.K., Lai W.H., Xiong Y.H. (2018). Quantum dot nanobead-based multiplexed immunochromatographic assay for simultaneous detection of aflatoxin B1 and zearalenone. Anal. Chim. Acta.

[24] Zhang Z.W., Tang X.Q., Wang D., Zhang Q., Li P.W., Ding X.X. (2015). Rapid On-Site Sensing Aflatoxin B1 in Food and Feed via a Chromatographic Time-Resolved Fluoroimmunoassay. PLoS ONE.

[25] Wang D., Zhang Z., Li P., Zhang Q., Zhang W. (2016). Time-resolved fluorescent immunochromatography of aflatoxin b1 in soybean sauce: A rapid and sensitive quantitative analysis. Sensors.

[26] Foubert A., Beloglazova N.V., De Saeger S. (2017). Comparative study of colloidal gold and quantum dots as labels for multiplex screening tests for multi-mycotoxin detection. Anal. Chim. Acta.

[27] Xie Q.Y., Wu Y.H., Xiong Q.R., Xu H.Y., Xiong Y.H., Liu K., Jin Y., Lai W.H. (2014). Advantages of fluorescent microspheres compared with colloidal gold as a label in immunochromatographic lateral flow assays. Biosens. Bioelectron..

[28] Wu C.H., Hu L.M., Xia J., Xu G.M., Luo K., Liu D.F., Duan H., Cheng S., Xiong Y.H., Lai W.H. (2017). Comparison of immunochromatographic assays based on fluorescent microsphere and quantum-dot submicrobead for quantitative detection of aflatoxin M1 in milk. J. Dairy Sci..

[29] Li S.J., Sheng W., Wen W., Gu Y., Wang J.P., Wang S. (2018). Three kinds of lateral flow immunochromatographic assays based on the use of nanoparticle labels for fluorometric determination of zearalenone. Microchim. Acta.

[30] Hu L.M., Luo K., Xia J., Xu G.M., Wu C.H., Han J.J., Zhang G.G., Liu M., Lai W.H. (2017). Advantages of time-resolved fluorescent nanobeads compared with fluorescent submicrospheres, quantum dots, and colloidal gold as label in lateral flow assays for detection of ractopamine. Biosens. Bioelectron..

[31] Luo K., Hu L., Guo Q., Wu C., Wu S., Liu D., Xiong Y.H., Lai W. (2017). Comparison of 4 label-based immunochromatographic assays for the detection of *Escherichia coli O157: H7* in milk. J. Dairy Sci..

[32] Wang J., Chang X.X., Zuo X.W., Liu H.B., Ma L.C., Li H.J., Tao X.Q. (2019). A Multiplex Immunochromatographic Assay Employing Colored Latex Beads for Simultaneously Quantitative Detection of Four Nitrofuran Metabolites in Animal-Derived Food. Food Anal. Methods.

[33] Chang X.X., Zhang Y.Q., Liu H.B., Tao X.Q. (2020). A quadruple-label time-resolved fluorescence immunochromatographic assay for simultaneous quantitative determination of three mycotoxins in grains. Anal. Methods.

[34] (2016). GB 5009.22-2016. National Food Safety Standards of the People’s Republic of China, Determination of Aflatoxin B and G in Foods.

[35] Liu B., Wang L.L., Tong B., Zhang Y., Sheng W., Pan M.F., Wang S. (2016). Development and comparison of immunochromatographic strips with three nanomaterial labels: Colloidal gold, nanogold-polyaniline-nanogold microspheres (GPGs) and colloidal carbon for visual detection of salbutamol. Biosens. Bioelectron..

[36] Chen Y.Q., Chen Q., Han M.M., Zhou J.Y., Gong L., Niu Y.M., Zhang Y., He L.D., Zhang L.Y. (2016). Development and optimization of a multiplex lateral flow immunoassay for the simultaneous determination of three mycotoxins in corn, rice and peanut. Food Chem..

[37] Sun S.J., Zheng P.M., Zhao S.J., Liu H.B., Wang Z.P., Peng T., Wang J.Y., Yao K., Wang S.H., Zeng Y.Y. (2018). Time-resolved fluorescent immunochromatographic assay-based on three antibody labels for the simultaneous detection of aflatoxin B1 and zearalenone in Chinese herbal medicines. Food Addit. Contam. A.

[38] Majdinasab M., Sheikh-Zeinoddin M., Soleimanian-Zad S., Li P.W., Zhang Q., Li X., Tang X.Q., Li J. (2015). A reliable and sensitive time-resolved fluorescent immunochromatographic assay (TRFICA) for ochratoxin A in agro-products. Food Control.

[39] Xu W., Xiong Y.H., Lai W.H., Xu Y., Li C.M., Xie M.Y. (2014). A homogeneous immunosensor for AFB_1_ detection based on FRET between different-sized quantum dots. Biosens. Bioelectron..

[40] Liu J.W., Lu C.C., Liu B.H., Yu F.Y. (2016). Development of novel monoclonal antibodies-based ultrasensitive enzyme-linked immunosorbent assay and rapid immunochromatographic strip for aflatoxin B1 detection. Food Control.

[41] Xue Z.H., Zhang Y.X., Yu W.C., Zhang J.C., Wang J.Y., Wan F., Kim Y., Liu Y.D., Kou X.H. (2019). Recent advances in aflatoxin B1 detection based on nanotechnology and nanomaterials—A review. Anal. Chim. Acta.

[42] Li X., Li P., Zhang Q., Li R., Zhang W., Zhang Z., Ding X., Tang X. (2013). Multi-component immunochromatographic assay for simultaneous detection of aflatoxin B1, ochratoxin A and zearalenone in agro-food. Biosens. Bioelectron..

