# Supplementary material for: Comparative Study of Time-Resolved Fluorescent Nanobeads, Quantum Dot Nanobeads and Quantum Dots as Labels in Fluorescence Immunochromatography for Detection of Aflatoxin B1 in Grains
Source: Biomolecules. 2020 Apr 9;10(4):575. doi: 10.3390/biom10040575 (PMC7226082; doi:10.3390/biom10040575)
Supplement: Supplementary file 1 [file biomolecules-10-00575-s001.pdf]

Comparative Study of Time-Resolved Fluorescent Nanobeads, Quantum Dot Nanobeads and Quantum Dots as Labels in Fluorescence Immunochromatography for Detection of Aflatoxin B_1 in Grains

Xin Wang [1,†], Xuan Wu [2,†], Zhisong Lu [3] and Xiaoqi Tao [1,*]

1 College of Food Science, Southwest University, Chongqing 400715, China; wx2018@email.swu.edu.cn
2 Chongqing Animal Disease Prevention and Control Center, Chongqing 401120, China; lzpzena@swu.edu.cn
3 Institute for Clean Energy & Advanced Materials, School of Materials & Energy, Southwest University, Chongqing 400715, China; zslu@swu.edu.cn
* Correspondence: taoxiaoqi@swu.edu.cn; Tel.: +86-18306008102
† These authors contributed equally to this work.

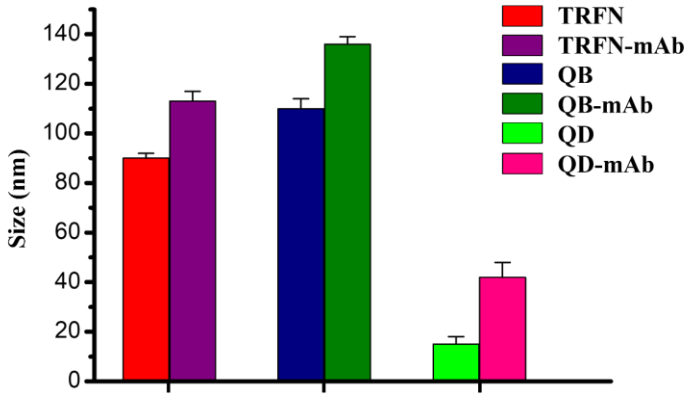

Figure S1. Particles size of three FICA labels.

1 Optimization of the three label-based FICA parameters

1.1 Optimization of the coupling pH

The labeling pH was one of the key factors to develop fluorescent probes, because it could influence activation of antibody and coupling efficiency. The fluorescent probes (TRFN probes, QB and QD probes) were prepared via EDC/NHS system to coupling amino group of antibody and carboxyl group of fluorescent nanoparticles. The activation of EDC was highest when the pH in a neutral acidic environment (ranged from 4.5-7.2)[1]. The Figure S2 indicated that inhibition rate of TRFN-FICA enhanced with the increase of pH, and decreased at pH 7.0. Therefore, labeling pH 7.0 was the optimal pH for coupling with TRFN. According to the same reason, pH 6.0 and pH 7.0 was the optimal labeling pH for the QB-mAb and QD-mAb.

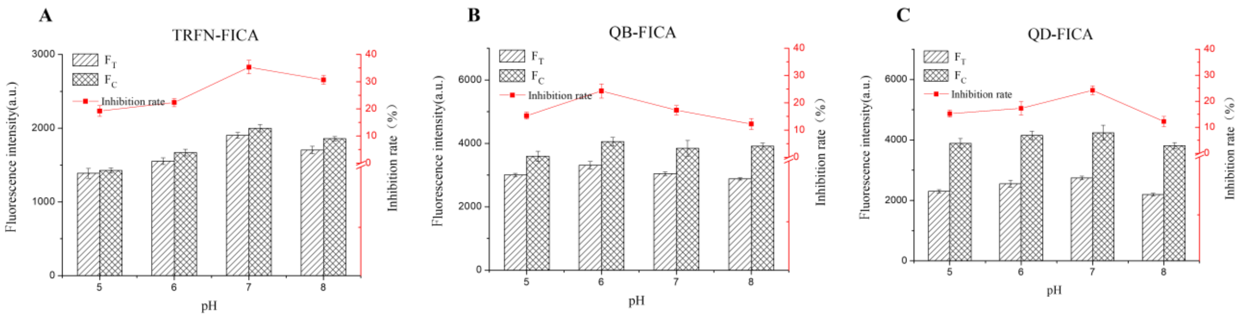

Figure S2. Optimization of coupling pH in preparing labeled-mAb probes: **(A)** TRFN-mAb; **(B)** QB-mAb; **(C)** QD-mAb.

1.2 Optimization of the concentration of anti-AFB_1-mAb in preparing labeled-mAb probes

The concentration of anti-AFB1-mAb was also a critical element for coupling with the labels. When the amount of the antibody was too small, there were rare coupled antibody on the excess fluorescent nanoparticles, and the limit structure of the antibody involved in the immune reaction was reduced; Conversely, it would be influenced the efficiency of fluorescence intensity when the amount of the antibody was too large. We analyzed the three FICAs labeled with different amounts of anti-AFB_1-mAb by studying the fluorescence intensity and competitive inhibition ratio of the *T* lines. Figure S3 indicated that the *T* line fluorescence intensity of TRFN-FICA gradually increased with the concentration of anti-AFB_1-mAb increases, and the *C* line signal intensity keeping stable because the amount of chicken IgY was constant. The optimum concentration of the established TRFN-FICA was 3 μg mL^{-1} of coating antigen. The optimum concentration of anti-AFB_1-mAb was 4.5 μg mL^{-1}, 4.5 μg mL^{-1} for QB-FICA and QD-FICA, respectively (Figure S3).

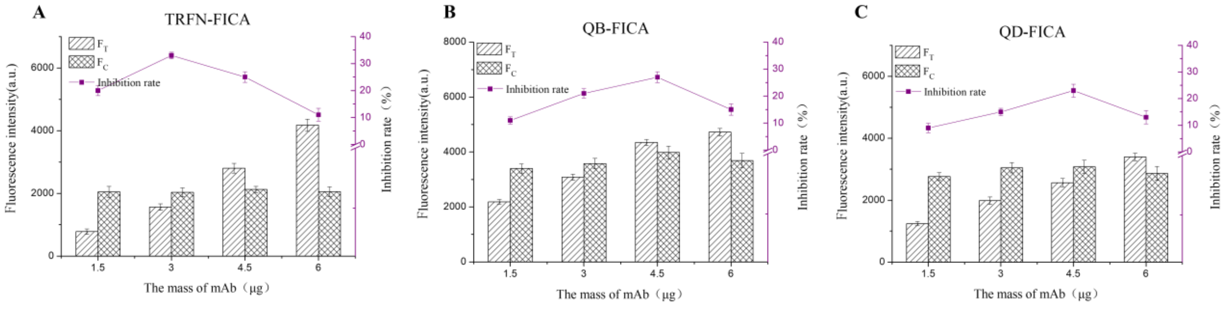

Figure S3. Optimization of the concentration of anti-AFB_1-mAb in preparing labeled-mAb probes: **(A)** TRFN-mAb; **(B)** QB-mAb; **(C)** QD-mAb.

1.3 Optimization of the concentration of AFB_1-CMO-BSA on T line

To achieve the best analytical performance of FICA, the concentration of antigen (AFB_1-CMO-BSA) coating on the NC membrane were investigated. As for TRFN-FICA, with increasing concentration of antigen, the competitive inhibition rate gradually decreased, a maximum competitive inhibition rate of 37.33% was observed in the coating concentration of 0.3 μg mL^{-1}, in which fluorescence intensity were also in proper situation (Figure S4). Therefore, the optimum

amounts of coating antigen were 0.30 μg mL^{-1}, 0.65μg mL^{-1} and 0.65μg mL^{-1} for TRFN-FICA, QB-FICA and QD-FICA, respectively.

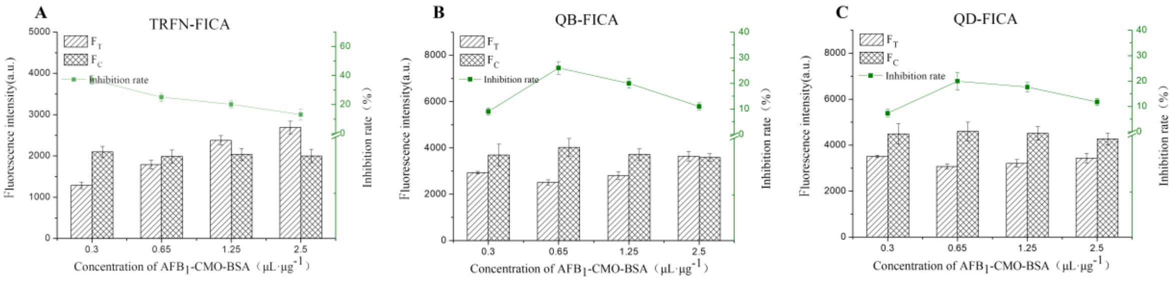

Figure S4. Optimization of the concentration of AFB_1-CMO-BSA for fluorescent immunochromatography: **(A)** TRFN-FICA; **(B)** QB-FICA; **(C)** QD-FICA.

1.4 Optimization of the immunochromatography reaction time

Point-of-care testing has high requirements for efficiency, and immunochromatographic methods have great advantages in this regard. However, when the reaction time is too short, the reaction is not complete, and fluorescence intensity is too low. Conversely, when the reaction time is too long, the efficiency is ignored. Therefore, appropriate reaction time not only ensures the accuracy of the detection but also takes into account of the efficiency, and Figure S5 reveals that the optimum immunochromatography duration were 25 min, 30 min and 35 min for TRFN-FICA, QD-FICA and QB-FICA, respectively.

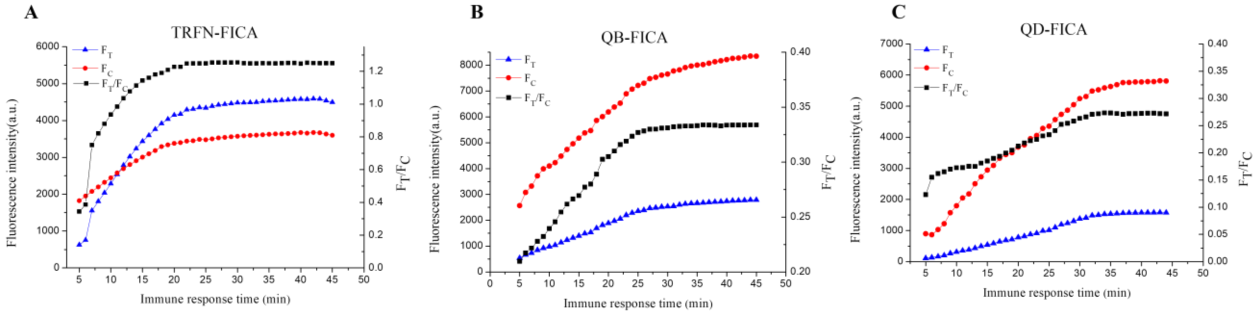

Figure S5. Immunoreaction dynamics of the three FICAs: **(A)** TRFN-FICA; **(B)** QB-FICA; **(C)** QD-FICA.

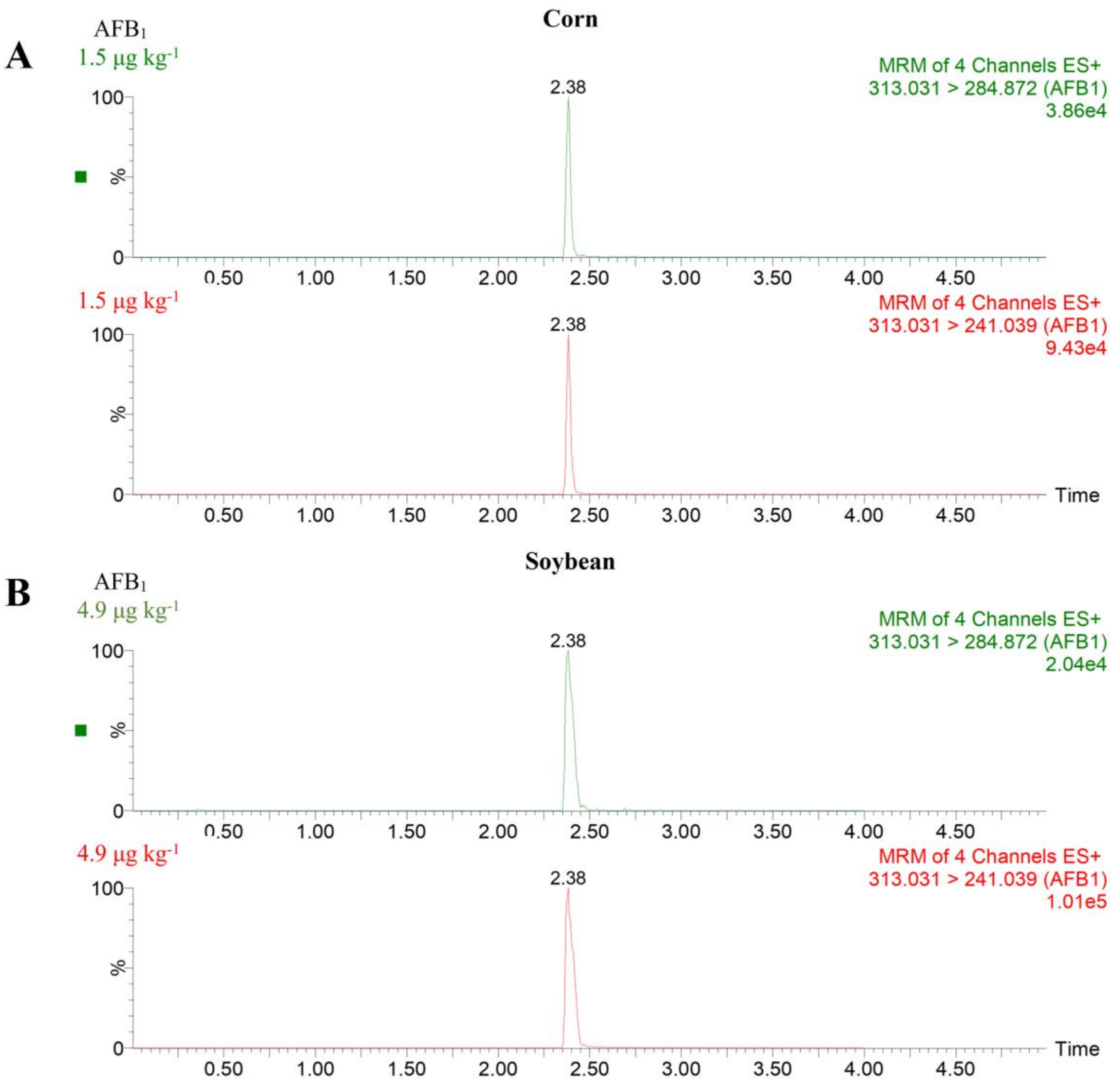

Figure S6. The representative mass chromatograms (lowest**(A)** and highest**(B)** concentrations of AFB_1) in real samples.

Table S1. Cross Reactivity (CR) of analytes with antibody detected by FICAs

CR（%）	AFB_1	AFB_2	AFM_1	AFG_1	AFG_2	OTA	DON	ZEN
TRFN-FICA	100%	18.15%	11.63%	5.42%	4.22%	<0.01%	<0.01%	<0.01%
QB-FICA	100%	18.54%	15.86%	5.33%	4.78%	<0.01%	<0.01%	<0.01%
QD-FICA	100%	19.96%	16.23%	6.20%	5.03%	<0.01%	<0.01%	<0.01%

Reference

1. Hu, L.M.; Luo, K.; Xia, J.; Xu, G.M.; Wu, C.H.; Han, J.J.; Zhang, G.G.; Liu, M.; Lai, W.H. Advantages of time-resolved fluorescent nanobeads compared with fluorescent submicrospheres, quantum dots, and colloidal gold as label in lateral flow assays for detection of ractopamine. *Biosens. & Bioelectron.* **2017**, *91*, 95-103.
